# Understanding the impact of COVID-19 on antibiotic use in Canadian primary care: a matched-cohort study using EMR data

**DOI:** 10.1186/s13756-024-01434-0

**Published:** 2024-07-12

**Authors:** Rachael Morkem, Glenys Smith, Braden Knight, Sabrina T. Wong, David Barber

**Affiliations:** 1 Department of Family Medicine, Queen’s University, 220 Bagot St., Kingston, ON K7L 5E9 Canada; 2 Public Health Agency of Canada, Ottawa, ON Canada; 3 Centre for Health Services and Policy Research and School of Nursing, University of British Columbia, Vancouver, Canada

**Keywords:** Primary care data, Antibiotic prescribing, Antimicrobial stewardship, Respiratory tract infection

## Abstract

**Background:**

Inappropriate or overuse of antibiotic prescribing in primary care highlights an opportunity for antimicrobial stewardship (AMS) programs aimed at reducing unnecessary use of antimicrobials through education, policies and practice audits that optimize antibiotic prescribing. Evidence from the early part of the pandemic indicates a high rate of prescribing of antibiotics for patients with COVID-19. It is crucial to surveil antibiotic prescribing by primary care providers from the start of the pandemic and into its endemic stage to understand the effects of the pandemic and better target effective AMS programs.

**Methods:**

This was a matched pair population-based cohort study that used electronic medical record (EMR) data from the Canadian Primary Care Sentinel Surveillance Network (CPCSSN). Participants included all patients that visited their primary care provider and met the inclusion criteria for COVID-19, respiratory tract infection (RTI), or non-respiratory or influenza-like-illness (negative). Four outcomes were evaluated (a) receipt of an antibiotic prescription; (b) receipt of a non-antibiotic prescription; (c) a subsequent primary care visit (for any reason); and (d) a subsequent primary care visit with a bacterial infection diagnosis. Conditional logistic regression was used to evaluate the association between COVID-19 and each of the four outcomes. Each model was adjusted for location (rural or urban), material and social deprivation, smoking status, alcohol use, obesity, pregnancy, HIV, cancer and number of chronic conditions.

**Results:**

The odds of a COVID-19 patient receiving an antibiotic within 30 days of their visit is much lower than for patients visiting for RTI or for a non-respiratory or influenza-like-illnesses (AOR = 0.08, 95% CI[0.07, 0.09] compared to RTI, and AOR = 0.43, 95% CI[0.38, 0.48] compared to negatives). It was found that a patient visit for COVID-19 was much less likely to have a subsequent visit for a bacterial infection at all time points.

**Conclusions:**

Encouragingly, COVID-19 patients were much less likely to receive an antibiotic prescription than patients with an RTI. However, this highlights an opportunity to leverage the education and attitude change brought about by the public health messaging during the COVID-19 pandemic (that antibiotics cannot treat a viral infection), to reduce the prescribing of antibiotics for other viral RTIs and improve antibiotic stewardship.

**Supplementary Information:**

The online version contains supplementary material available at 10.1186/s13756-024-01434-0.

## Background

The value of antimicrobial therapies is threatened by the growth and spread of antibiotic resistant bacteria, in part, due to the inappropriate/overuse of antibiotics [1]. In Canada, and throughout most developed countries, the majority of antibiotics are prescribed in the community. As such, it is essential to understand antibiotic prescribing practices in the primary care setting [2–4].

Several Canadian studies have shown that there is significant opportunity to reduce potentially inappropriate antibiotic prescribing in primary care [4–8]. A 2019 study using Canadian electronic medical record (EMR) data found significant variation in the proportion of acute respiratory tract infections (RTI) treated with antibiotics, with the top quartile of prescribers treating the common cold with an antibiotic more than half the time [5].

Inappropriate or overuse of antibiotic prescribing in primary care highlights an opportunity for antimicrobial stewardship (AMS) programs aimed at reducing unnecessary use of antimicrobials through education, policies and practice audits that optimize antibiotic prescribing [9, 10]. Prior to the onset of the COVID-19 pandemic, antibiotic prescribing rates in the community were stable. A report from the Public Health Agency of Canada described that between 2014 and 2018 antibiotic prescribing by family physicians and general practitioners only dropped by 3%, from 428.3 to 416.2 prescriptions per 1000 inhabitants [4]. However, the spread of the COVID-19 virus in early 2020 led to unprecedented changes in not only the delivery of healthcare but also in the transmission of communicable pathogens [11, 12]. Antibiotic prescribing in primary care during the pandemic significantly decreased, most notably for respiratory tract infections [12–17].

With the emergence of a novel virus, it is hypothesized that this inappropriate prescribing would also be seen amongst patients with COVID-19. Evidence from the early part of the pandemic indicates a high rate of prescribing of antibiotics for patients with COVID-19 [18]. However, it is unclear if this inappropriate prescribing was sustained. As such, it is crucial to surveil antibiotic prescribing by primary care practitioners from the start of the pandemic and into its endemic stage to understand the effects of the pandemic and better target effective AMS programs.

The objective of this study was to compare antibiotic prescribing and healthcare utilization between COVID-19 positive patients to those with a) respiratory tract infection (RTI); and b) COVID-19 negative patients.

## Methods

### Data source and study design

This was a matched pair population-based cohort study that used electronic medical record (EMR) data from the Canadian Primary Care Sentinel Surveillance Network (CPCSSN). CPCSSN is a federated network of fourteen practice-based research and learning networks (PBRLN) across Canada. Primary care providers, known as sentinels, share clinical data on their patients with CPCSSN to form a national data repository of patient clinical records. The CPCSSN repository has data on over 1.8 million patients, contributed by over 1,500 primary care providers from British Columbia, Alberta, Manitoba, Ontario, Quebec, Nova Scotia and Newfoundland. While patients represented within this pan-Canadian data repository are older and have more females than the overall Canadian population, the data repository is representative of Canadians who visit primary care [19].

This study excluded clinical data from Manitoba and Quebec as we were unable to classify COVID-19 cases in the data from these two PBLRNs. In these two provinces the lab results confirming a COVID-19 infection were recorded almost exclusively as PDF documents, which are not available (at this time) within the CPCSSN database.

### Participants

Participants included all patients that visited their primary care provider and met the inclusion criteria for COVID-19, RTI, or negative (see Additional file 1 for case definitions). To be included a patient had to have a documented birthyear and sex.

#### COVID-19 encounter

A patient’s visit was included as a COVID-19 primary care visit if there was a diagnosis and/or lab-confirmation of the COVID-19 virus during a primary care visit between April 2020 and December 2021. A patient could only be included as a COVID-19 visit once (incident case = index event).

#### RTI encounter

A patient’s visit was included as an RTI primary care visit if they had a diagnosis of RTI during a primary care visit between April 2020 and December 2021. Encounters were only included if there was no mention of COVID-19 in any record associated with the encounter date.

#### Negative encounter

A patient’s visit was included as a ‘negative’ primary care visit if they had a visit with no diagnostic or lab data corresponding to a condition with respiratory symptoms (see Additional file 1 for list of exclusion criteria). Encounters were only included if there was no mention of COVID-19 in any record associated with the encounter date.

Each group was mutually exclusive. Other than the COVID-19 visit group, a patient visit was eligible to be included once every six months. A patient visit (for RTI or negative) within six months of a previous visit (for RTI or negative, respectively) were not eligible for inclusion as the outcome was evaluated up to 180 days after the index event.

We also calculated the proportion of visits that had COVID-19 documentation within the EMR out of all visits during the study time period.

### Outcome

Four outcomes were evaluated at four follow up intervals (30 days, 60 days, 90 days, and 180 days): (a) receipt of an antibiotic prescription; (b) receipt of a non-antibiotic prescription; (c) a subsequent primary care visit (for any reason); and (d) a subsequent primary care visit with a bacterial infection diagnosis (see Additional file 2 for a full description of how each outcome was defined).

### Matching

In order to compare the COVID-19 visits with each of the comparison groups the COVID-19 subjects were matched (1:1) to an RTI visit and a negative visit on the following covariates: index month (month of diagnosis), age group (0–18, 19–39, 40–64, 65 +), sex (male, female), and province (British Columbia, Alberta, Ontario, Nova Scotia, and Newfoundland).

### Covariates

Patient age was determined at time of the index visit. Rural or urban patient locations were determined based on the second digit of the postal code, which indicates whether the patient lives in an urban (1–9) or rural area (0), as defined by Canada Post delivery areas. If a patient was missing a postal code their rural urban status was classified using the postal code of the clinic.

A material and social deprivation index (Pampolon), derived from a linkage between postal code and census data, was used as a proxy for socioeconomic status [20]. Measures of material and social deprivation were derived from the postal code using the Statistics Canada Postal Code Conversion File, along with the material and social deprivation index [20]. This index uses socioeconomic indicators from the census, including education, employment, and income (the material component), as well as marital status and family structure (the social component), to assign scores to dissemination areas (DA) in the form of population quintiles. Mean imputation was used to compute a material or social deprivation score for patients that were missing this covariate.

CPCSSN validated case definitions were used to classify a patient’s comorbidity status, specifically for the following conditions: chronic kidney disease (CKD), chronic obstructive pulmonary disease (COPD), dementia, depression, diabetes, epilepsy, hypertension, osteoarthritis, Parkinson’s disease, dyslipidemia, and asthma [21–24]. A patient was classified as obese if there was a recorded BMI observation ≥ 30 kg/m^2^.

A patient was classified as a smoker, non-smoker, past smoker or unknown based on smoking risk factor records. Similarly, alcohol use was classified as yes, no, or unknown based on alcohol risk factor records.

A patient was classified as pregnant, HIV positive, or has cancer if they had an associated ICD-9 code during the study period (March 2020 to December 2022). The ICD-9 codes used to classify patients for each of these covariates are described in Additional file 2.

### Analysis

Conditional logistic regression, a specialized type of regression appropriate for individually matched case–control data, was used to evaluate the association between COVID-19 and each of the four outcomes. Each model was adjusted for location (rural or urban), material and social deprivation, smoking status, alcohol use, obesity, pregnancy, HIV, cancer and number of chronic conditions. As this was an exploratory analysis and there were no a priori hypotheses, all covariates were kept in the fully adjusted model.

The data analyses were completed using SAS© 9.4.

## Results

Between April 1^st^, 2020, and December 31^st^, 2021, there were 855,434 patients that had at least one visit with a participating primary care provider. Of these patients, there were 19,890 that visited their primary care provider for a COVID-19 infection during the study period. 1.57% of all patient visits had documentation of COVID-19. In comparison to patients visiting primary care for other reasons, COVID-19 patients were significantly more likely to be younger (34.7% of COVID-19 patients were young adults 19–39 years, compared to 23.3% of all other patients), more urban (90.5% compared to 82.5%) and have less comorbidities (56.8% have ≥ 1 comorbidity compared to 64.2%) (Table 1). Of the 19,890 COVID-19 visits 19,020 were matched to an RTI visit, and all 19,890 were matched to a negative visit. 17.5% of patients were missing a social and material index and were assigned a value using mean imputation. Table 2 compares the demographic and clinical characteristics of the COVID-19 group to each matched control group.

**Table 1:** Comparison of demographic and clinical characteristics of patients who visited primary care for any reason to patients who visited for COVID-19, RTI, or a non-respiratory illness (negative)

	All patients	COVID-19 patients	*p* value	All patients	RTI	*p* value	All patients	Negative^‡^	*p* value
**Population size, n**	855,434	19,890		855,434	18,773		855,434	19,890	
**Female**	478,381 (56.0)	10,994 (55.3)	0.0477	478,381 (56.0)	10,591 (56.4)	0.2326	478,381 (56.0)	10,994 (55.3)	0.0477
**Age groups**									
0–18	145,791 (17.5)	3,826 (19.2)	< .0001	145,791 (17.5)	3,791 (20.2)	< .0001	145,791 (17.5)	3,826 (19.2)	< .0001
19–39	193,573 (23.3)	6,894 (34.7)		193,573 (23.3)	6,178 (32.9)		193,573 (23.3)	6,894 (34.7)	
40–64	285,520 (34.3)	7,205 (36.2)		285,520 (34.3)	6,845 (36.5)		285,520 (34.3)	7,205 (36.32)	
65 +	206,266 (24.8)	1,965 (9.9)		206,266 (24.8)	1,959 (10.4)		206,266 (24.8)	1,965 (9.9)	
**Province**									
British Columbia	68,756 (8.0)	2,036 (10.2)	< .0001	68,756 (8.0)	2,010 (10.7)	< .0001	68,756 (8.0)	2,036 (10.2)	< .0001
Alberta	195,963 (22.9)	7,055 (35.5)		195,963 (22.9)	6,926 (36.9)		195,963 (22.9)	7,055 (35.5)	
Ontario	541,006 (63.2)	10,598 (53.3)		541,006 (63.2)	9,645 (51.4)		541,006 (63.2)	10,598 (53.3)	
Nova Scotia	46,344 (5.4)	174 (0.9)		46,344 (5.4)	174 (0.93)		46,344 (5.4)	174 (0.9)	
Newfoundland	3,365 (0.4)	27 (0.1)		3,365 (0.4)	18 (0.10)		3,365 (0.4)	27 (0.1)	
**Location**									
Urban	679,043 (82.5)	17,450 (90.5)	< .0001	679,043 (82.5)	16,912 (90.1)	< .0001	679,043 (82.5)	16,941 (86.6)	< .0001
**Social deprivation**									
Most deprived (4,5)	298,003 (34.8)	6,477 (32.6)	< .0001	298,003 (34.8)	5,910 (31.5)	< .0001	298,003 (34.8)	7,149 (35.9)	0.0012
**Material deprivation**									
Most deprived (4,5)	239,374 (28.0)	4,727 (23.8)	< .0001	239,374 (28.0)	4,330 (23.1)	< .0001	239,374 (28.0)	6,251 (31.4)	< .0001
**Pregnant**	8,350 (0.98)	312 (1.6)	< .0001	8,350 (0.98)	212 (1.13)	0.0350	8,350 (1.0)	245 (1.2)	0.0003
**HIV infection**	663 (0.08)	50 (0.2)	< .0001	663 (0.08)	11 (0.06)	0.3558	663 (0.08)	7 (0.04)	0.0329
**Cancer**	76,581 (8.9)	1,466 (7.4)	< .0001	76,581 (8.95)	1,972 (10.50)	< .0001	76,581 (8.9)	1,823 (9.2)	0.2981
**Smoking**									
Non-smoker	270,512 (31.6)	7,296 (36.7)	< .0001	270,512 (31.6)	7,466 (39.8)	< .0001	270,512 (31.6)	8,552 (43.0)	< .0001
Past smoker	77,108 (9.0)	1,441 (7.2)		77,108 (9.0)	1,669 (8.9)		77,108 (9.0)	1,592 (8.0)	
Smoker	101,384 (11.8)	2,020 (10.2)		101,384 (11.8)	2,111 (11.2)		101,384 (11.8)	2,270 (11.4)	
Unknown	406,430 (47.5)	9,133 (45.9)		406,430 (47.5)	7,527 (40.1)		406,430 (47.5)	7,476 (37.6)	
**Alcohol use**									
Yes	222,239 (26.0)	5,506 (27.7)	0.0589	222,239 (26.0)	5,244 (27.9)	0.7579	222,239 (26.0)	5,482 (27.6)	0.0003
No	69,735 (8.2)	1,810 (9.1)		69,735 (8.2)	1,921 (10.2)		69,735 (8.2)	2,224 (11.2)	
Unknown	563,460 (65.9)	12,574 (63.2)		563,460 (65.9)	11,608 (61.8)		563,460 (65.9)	12,184 (61.3)	
**Obese**	178,214 (20.8)	4,314 (21.7)	0.0033	178,214 (20.8)	4,316 (22.99)	< .0001	178,214 (20.8)	3,857 (19.4)	< .0001
**Chronic conditions**									
0	306,017 (35.8)	8,594 (43.2)	< .0001	306,017 (35.8)	5,392 (28.7)	< .0001	306,017 (35.8)	7,757 (39.0)	< .0001
≥ 1	549,417 (64.2)	11,296 (56.8)		549,417 (64.2)	13,381 (71.3)		549,417 (64.2)	12,133 (61.0)

**Table 2:** Comparison of demographic and clinical characteristics of patients with COVID-19 to each matched control (RTI and Negative)

	COVID-19 patients	RTI	*p* value	COVID-19 patients	Negative^‡^	*p* value
**Population size, n**	18,773	18,773		19,890	19,890	
**Location**						
Urban	16,948 (90.3)	16,912 (90.1)	0.5324	17,993 (90.5)	17,228 (86.6)	< .0001
**Social deprivation**						
Most deprived (4,5)	5,959 (31.7)	5,910 (31.5)	0.5865	6,477 (32.6)	7,149 (35.9)	< .0001
**Material deprivation**						
Most deprived (4,5)	4,434 (23.6)	4,330 (23.1)	0.2045	4,727 (23.8)	6,251 (31.4)	< .0001
**Pregnant**	301 (1.6)	212 (1.1)	< .0001	312 (1.6)	245 (1.2)	0.0043
**HIV infection**	49 (0.3)	11 (0.06)	< .0001	50 (0.25)	7 (0.04)	< .0001
**Cancer**	1,400 (7.5)	1,972 (10.5)	< .0001	1,466 (7.4)	1,823 (9.2)	< .0001
**Smoking**						
Non-smoker	6,804 (36.2)	7,466 (39.8)	< .0001	7,296 (36.7)	8,552 (43.0)	< .0001
Past smoker	1,371 (7.3)	1,669 (8.9)		1,441 (7.2)	1,592 (8.0)	
Smoker	1,900 (10.1)	2,111 (11.2)		2,020 (10.2)	2,270 (11.4)	
Unknown	8,698 (46.3)	7,527 (40.1)		9,133 (45.9)	7,476 (37.6)	
**Alcohol use**						
Yes	5,086 (27.1)	5,244 (27.9)	0.6646	5,506 (27.7)	5,482 (27.6)	0.0002
No	1,714 (9.1)	1,921 (10.2)		1,810 (9.1)	2,224 (11.2)	
Unknown	11,973 (63.8)	11,608 (61.8)		12,574 (63.2)	12,184 (61.3)	
**Obese**	4,095 (21.8)	4,316 (23.0)	0.0062	4,314 (21.7)	3,857 (19.4)	< .0001
**Chronic conditions**						
0	8,025 (42.7)	5,392 (28.7)	< .0001	8,594 (43.2)	7,757 (39.0)	< .0001
≥ 1	10,748 (57.2)	13,381 (71.3)		11,296 (56.8)	12,133 (61.0)	

**Fig. 1:**
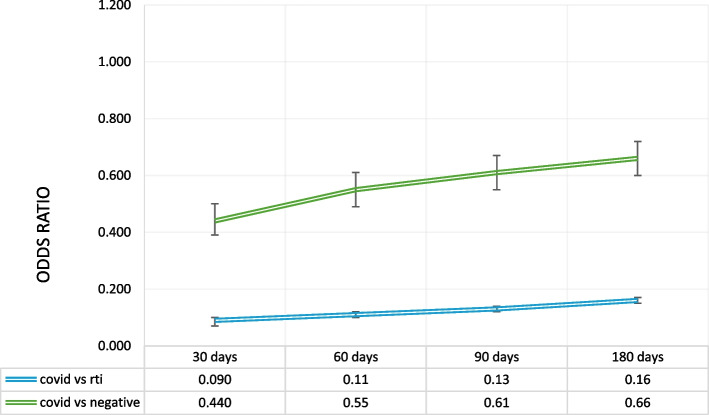
Conditional logistic regression for receipt of an antibiotic. ‡Adjusted for age, sex, location, social and material deprivation, smoking, alcohol use, obesity, pregnancy, HIV, cancer and chronic comorbidities

The unadjusted and adjusted odds ratios for each outcome are displayed in Table 3. Figure 1 exhibits the results of the conditional logistic regression analysis for receipt of an antibiotic prescription within 30 days, 60 days, 90 days, and 180 days of the index event. The adjusted odds of a COVID-19 patient receiving an antibiotic within 30 days of their visit is much lower than for patients visiting for RTI or for a non-respiratory or influenza-like-illnesses (AOR = 0.09, 95% CI[0.07,0.09] compared to RTI, and AOR = 0.43, 95% CI[0.38, 0.48] compared to negatives). This remained true across all time periods (Fig. 1).

**Table 3:** Unadjusted and adjusted odds ratios for: A. receipt of an antibiotic prescription; B. receipt of a non-antibiotic prescription; C. subsequent primary care visit; and D. subsequent primary care visit for bacterial infection

		**COVID-19: RTI**		**COVID-19: negative**	
		Unadjusted OR (95% CI)	Adjusted OR (95% CI)	Unadjusted OR (95% CI)	Adjusted OR (95% CI)
A. Receipt of an antibiotic prescription	30 days	0.09 (0.08, 0.10)	0.09 (0.07, 0.10)	0.44 (0.39, 0.49)	0.44 (0.39, 0.50)
	60 days	0.11 (0.10, 0.12)	0.11 (0.10, 0.12)	0.52 (0.47, 0.58)	0.55 (0.49, 0.61)
	90 days	0.13 (0.12, 0.14)	0.13 (0.12, 0.14)	0.58 (0.53, 0.64)	0.61 (0.55, 0.67)
	180 days	0.16 (0.15, 0.17)	0.16 (0.15, 0.17)	0.63 (0.58, 0.69)	0.66 (0.60, 0.72)
B. Receipt of non- antibiotic prescription	30 days	0.44 (0.42, 0.47)	0.44 (0.42, 0.47)	0.25 (0.24, 0.27)	0.26 (0.24, 0.27)
	60 days	0.51 (0.49, 0.54)	0.52 (0.49, 0.54)	0.35 (0.33, 0.36)	0.35 (0.34, 0.37)
	90 days	0.54 (0.52, 0.57)	0.54 (0.52, 0.57	0.41 (0.40, 0.43)	0.42 (0.40, 0.44)
	180 days	0.55 (0.53, 0.57)	0.55 (0.53, 0.58)	0.48 (0.46, 0.50)	0.48 (0.46, 0.51)
C. Subsequent visit	30 days	1.53 (1.45, 1.59)	1.59 (1.52, 1.67)	1.50 (1.44, 1.56)	1.52 (1.45, 1.58)
	60 days	1.28 (1.23, 1.34)	1.32 (1.26, 1.39)	1.34 (1.29, 1.40)	1.35 (1.29, 1.41)
	90 days	1.13 (1.08, 1.19)	1.17 (1.11, 1.22)	1.23 (1.18, 1.29)	1.24 (1.19, 1.30)
	180 days	0.93 (0.88, 0.97)	0.96 (0.91, 1.01)	1.00 (0.95, 1.05)	0.99 (0.95, 1.04)
D. Subsequent visit with bacterial infection	30 days	0.07 (0.06, 0.07)	0.07 (0.06, 0.07)	0.90 (0.84, 0.99)	0.97 (0.89, 1.06)
	60 days	0.08 (0.08, 0.09)	0.08 (0.07, 0.09)	1.00 (0.93, 1.08)	1.06 (0.98, 1.15)
	90 days	0.09 (0.08, 0.10)	0.09 (0.08, 0.10)	1.04 (0.96, 1.11)	1.10 (1.01, 1.18)
	180 days	0.11 (0.10, 0.11)	0.11 (0.10, 0.12)	1.07 (1.00, 1.14)	1.11 (1.04, 1.19)

**Fig. 2:**
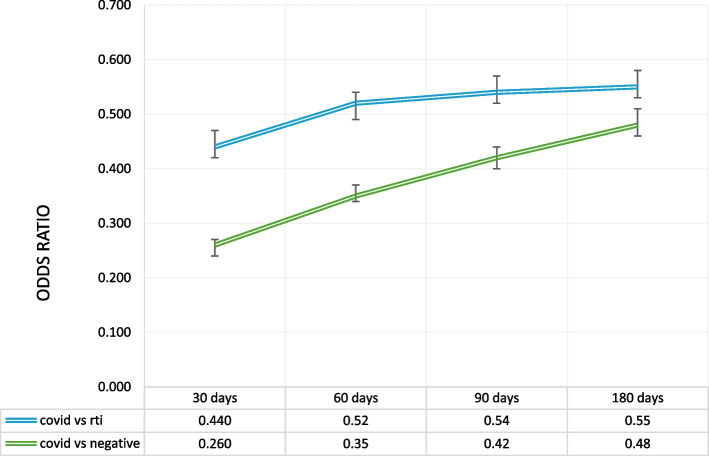
Conditional logistic regression for receipt of a non-antibiotic medication. ‡Adjusted for age, sex, location, social and material deprivation, smoking, alcohol use, obesity, pregnancy, HIV, cancer and chronic comorbidities

The conditional logistic regression analysis for receipt of a non-antibiotic prescription in the days following a visit revealed that a patient encounter with a COVID-19 code was much less likely to receive a prescription than a patient visit for RTI or a patient visit unrelated to respiratory or influenza-like illness (negative) (Fig. 2).

**Fig. 3:**
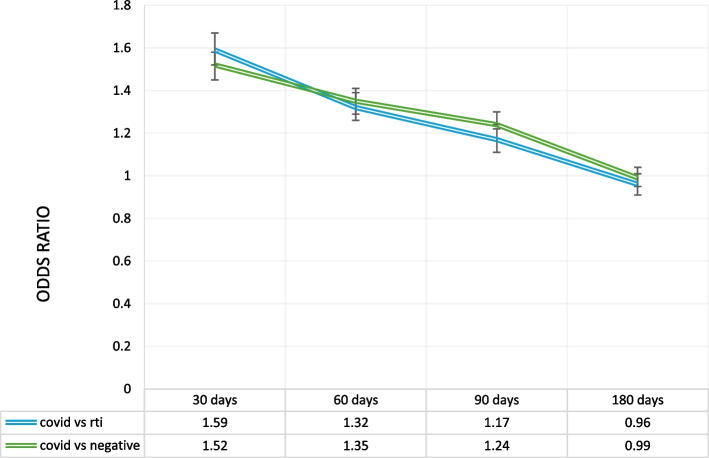
Conditional logistic regression for a subsequent primary care visit. ‡Adjusted for age, sex, location, social and material deprivation, smoking, alcohol use, obesity, pregnancy, HIV, cancer and chronic comorbidities

The third outcome evaluated using conditional logistic regression was a subsequent primary care visit and it was found that a patient presenting with COVID-19 was much more likely to have a subsequent visit within 30 days compared to a patient visiting for RTI (AOR = 1.61, 95% CI[1.53, 1.68], or a non-respiratory or non-influenza like-illness (AOR = 1.52, 95% CI[1.45, 1.58]) (Fig. 3). This increased likelihood remained true at 60 days (AOR = 1.33, 95% CI[1.27, 1.40] compared to RTI, and AOR = 1.35, 95% CI[1.29, 1.40] compared to negative), but by 180 days after the index visit patients with COVID-19 were as likely to have a subsequent visit as patients with RTI (90 day AOR = 1.17, 95% CI[1.14, 1.23], 180 day AOR = 0.96, 95% CI[0.91, 1.01]) or negative (90 day AOR = 1.23, 95% CI[1.18, 1.29], 180 day AOR = 0.99, 95% CI[0.94, 1.04].

**Fig. 4:**
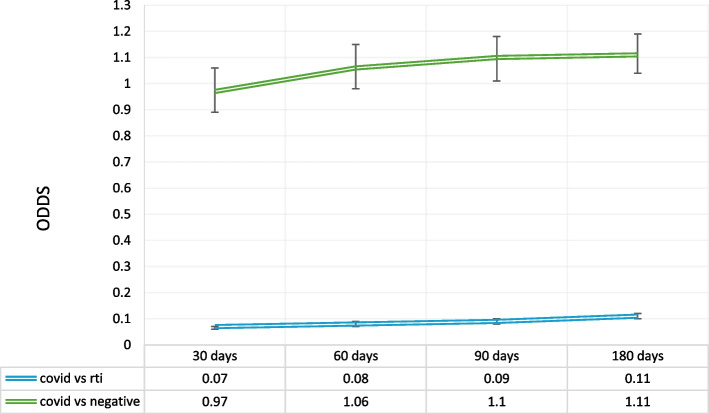
Conditional logistic regression for a subsequent primary care visit with a bacterial infection. ‡Adjusted for age, sex, location, social and material deprivation, smoking, alcohol use, obesity, pregnancy, HIV, cancer and chronic comorbidities

Lastly, we evaluated the likelihood of a subsequent visit for a bacterial infection (Fig. 4). It was found that a patient visit for COVID-19 was much less likely to have a subsequent visit for a bacterial infection at all time points, in comparison to patients visiting for RTI (30 day AOR = 0.07, 95% CI[0.06, 0.07], 60 day AOR = 0.08, 95% CI[0.07,0.09], 90 day AOR = 0.09, 95% CI[0.08, 0.10], 180 day AOR = 0.11, 95% CI[0.10, 0.11]).

## Discussion

Overall, in the first year and a half of the pandemic, there were a low number of primary care visits where COVID-19 was documented (1.57%). This low number of COVID-19 visits suggests that most patients with COVID-19 infections were either not seeking healthcare (mild cases) or potentially seeking healthcare at alternate settings such as COVID-19 assessment centres or emergency rooms (severe cases).

Similar to past work, we found patients that did visit primary care with a COVID-19 infection were much less likely to receive an antibiotic prescription. Two studies using Canadian pharmacy data also found that there has been an overall significant reduction in total outpatient antibiotic prescriptions issued during the COVID-19 pandemic [12, 24]. Integrating the results from the pharmacy data with the results from this study suggests that the reduction may be due to a change in the amount of circulating respiratory tract viruses, rather than a change in the propensity to prescribe for a respiratory virus. In addition, research shows that public messaging and social media has a significant influence on patient behaviour, such as medication seeking [25]. It is possible that the reduction in overall antibiotic prescribing was also due to effective public health awareness campaigns that antibiotics are not useful to treat viral infections like COVID-19. Nonetheless, these findings emphasize that there is room for improvement in reducing potentially inappropriate antibiotic prescribing for non-COVID-19 RTIs.

The observation that COVID-19 patients are more likely to have a subsequent visit within 60 days of their index event compared to RTI or negative patients may be due to the uncertainty and fear around the outcomes of a COVID-19 infection during the first 18 months of the pandemic, or that patients presenting to primary care with COVID-19 represent more severe or lingering cases that require a higher level of primary care compared to those that do not seek medical attention.

The finding that a patient with a COVID-19 visit is less likely to receive a non-antibiotic prescription than patient encounters for RTI or negative may reflect how the use of primary care visits changed due to the pandemic, with patients combining multiple needs into one visit because of public health measures and reduced access to primary care. A recent study evaluating reasons for primary care visits during the pandemic found that there was an increase in visits for anxiety but a decrease in visits for preventive care and chronic disease [26]. Patient encounters for RTI or non-respiratory illness (negative) during the COVID-19 pandemic may be more likely to be combining multiple health concerns into one appointment than encounters for COVID-19.

Reassuringly, this study reveals that patients presenting with COVID-19 are much less likely to have record of a subsequent bacterial infection in the 180 days following their COVID-19 visit, compared to RTI patients. In contrast, COVID-19 patients were as likely to have record of a bacterial infection within 30 days of their index event compared to patients visiting for a non-respiratory illness or health concern, and more likely to have record of a bacterial infection between 60 to 180 days following their COVID-19 visit compared to patients visiting for a non-respiratory illness or health concern. These findings suggest that in comparison to other respiratory viruses the COVID-19 virus does not increase a patient’s vulnerability to a bacterial infection, and COVID-19 patients are like other primary care patients seeking care for non-respiratory illness concerns.

### Limitations

There are several limitations to this study. First, the EMR data used in this study were collected for clinical and administrative purposes and may result in incomplete capture of information. Second, the clinical context for some of the diagnoses and outcomes evaluated in this study can be difficult to infer from secondary use of EMR data. Third, there is likely some misclassification as the ability to identify patients with COVID-19 during the first 18 months of the pandemic is dictated by testing procedures in each jurisdiction.

It is likely that some patients within the matched groups (RTI and negative) had COVID-19 infections during the study period but it was not indicated in the EMR of that patient. Furthermore, documentation patterns at the onset of the pandemic were variable and could lead to some misclassification. This may be particularly true for RTI encounters as it is uncertain how providers were documenting RTI during the initial years of the COVID-19 pandemic. However, attempts were made to minimize this misclassification by only including RTI encounters if there was no mention of COVID-19 in any records associated with that encounter date. There were also variable treatment protocols over the course of the early stages of the pandemic and this study is not able to account for different regimens at different institutions.

Neighborhood-level indicators of socioeconomic status were used, and this could also contribute to some misclassification.

## Conclusion

This study evaluated the primary healthcare services provided to patients with documented COVID-19 in comparison to patients presenting with RTI and non-respiratory conditions. Encouragingly, COVID-19 patients were much less likely to receive an antibiotic prescription than patients with an RTI. However, this highlights an opportunity to leverage the education and attitude change brought about by the public health messaging during the COVID-19 pandemic (that antibiotics cannot treat a viral infection), to reduce the prescribing of antibiotics for other viral RTIs and improve antibiotic stewardship. This has a direct impact on patient outcomes by reducing morbidity and mortality from antimicrobial resistant infections [9, 27].

### Supplementary Information

Additional file 1.

Additional file 2.

## Data Availability

The data used in this work was from the Canadian Primary Care Sentinel Surveillance Network (CPCSSN). It can be accessed via their website www.cpcssn.org.

## Abbreviations

AMS: Antimicrobial Stewardship
ATC: Anatomical Therapeutic Classification
CKD: Chronic Kidney Disease
COPD: Chronic Obstructive Pulmonary Disease
CPCSSN: Canadian Primary Care Sentinel Surveillance Network
DA: Dissemination Area
EMR: Electronic Medical Record
ICD-9: International Classification of Diseases – 9th version
AOR: Adjusted Odds Ratio
PBLRN: Practice Based Research and Learning Network
RTI: Respiratory Tract Infection
