# Supplementary material for: Understanding the impact of COVID-19 on antibiotic use in Canadian primary care: a matched-cohort study using EMR data
Source: Antimicrob Resist Infect Control. 2024 Jul 12;13:76. doi: 10.1186/s13756-024-01434-0 (PMC11242630; doi:10.1186/s13756-024-01434-0)
Supplement: Supplementary file 1 — Additional file 1. [file 13756_2024_1434_MOESM1_ESM.docx]

**Additional File 1**

COVID-19 Case Definition

A patient encounter was classified as a COVID-19 encounter if it met the following inclusion criteria:

| **Table** | **Codes** | **Result** |
| --- | --- | --- |
| Lab | 94309-2 | ‘Positive’ |
| Health Condition | O79.82 | -- |


Respiratory Tract Infection Case Definition (five syndromes)

A patient encounter was classified as an RTI encounter if it met the following inclusion and exclusion criteria:

| **Syndrome** | **Inclusion Codes** | **Tables** | **Exclusion Codes** |
| --- | --- | --- | --- |
| OM  *6months – 17 years (inclusive)* | 381  382 | Billing  Encounter Diagnosis  Health Conditions | 381.6  381.7  381.8  381.9 |
| Uncomplicated Pharyngitis | 034  463  464  462 | Billing  Encounter Diagnosis  Health Conditions | “Abscess” or “Mononucleosis” in text_orig field in any of the three tables. |
| Uncomplicated Sinusitis | 461 | Billing  Encounter Diagnosis  Health Conditions | 473 |
| URTI – Common Cold | 460  465 | Billing  Encounter Diagnosis  Health Conditions | None |
| Acute COPD and Bronchitis/Asthma (ie. Chronic lung condition exacerbations) | 466  491  492  493  496 | Billing  Encounter Diagnosis  Health Conditions | COPD in CASE DEFINITION TABLE |

‘Negative’ Case Definition

A patient encounter was classified as a negative encounter if it met the following criteria:

1. did not meet the case definitions for COVID-19, influenza or RTI; or
2. did not have a billing or encounter diagnosis for O79.32 (SARS-COV-2)
