# Supplementary material for: Understanding the impact of COVID-19 on antibiotic use in Canadian primary care: a matched-cohort study using EMR data
Source: Antimicrob Resist Infect Control. 2024 Jul 12;13:76. doi: 10.1186/s13756-024-01434-0 (PMC11242630; doi:10.1186/s13756-024-01434-0)
Supplement: Supplementary file 2 — Additional file 2. [file 13756_2024_1434_MOESM2_ESM.docx]

**Additional File 2**

Outcome definitions

1. Prescription for an antibiotic – defined as any oral medication corresponding to Anatomical Therapeutic Classification (ATC) code J01%
2. Prescription for a non-antibiotic – defined as any oral medication not corresponding to ATC code J01%.
3. Subsequent visit – defined as
4. Subsequent visit with a documentation of a bacterial infection – defined as any visit with one of the ICD-9 codes listed in the table below found in the Billing table, the Encounter Diagnosis table, or the Health Conditions table.

Bacterial Infection Diagnostic Codes

| 1 | 16 | 38.11 | 82 | 98.1 | 320.9 | 424.99 | 568 | 614.5 | 682.1 | 730.24 | 996.62 |
| --- | --- | --- | --- | --- | --- | --- | --- | --- | --- | --- | --- |
| 1 | 16.1 | 38.12 | 82.3 | 98.13 | 324 | 463 | 569.5 | 614.6 | 682.2 | 730.26 | 996.64 |
| 2 | 16.2 | 38.2 | 82.4 | 98.15 | 324.1 | 464 | 569.61 | 616 | 682.3 | 730.27 | 996.66 |
| 2.2 | 16.3 | 38.4 | 82.41 | 98.16 | 324.9 | 464.1 | 572 | 616.3 | 682.4 | 730.28 | 998.5 |
| 2.9 | 16.4 | 38.42 | 82.49 | 98.2 | 326 | 474.01 | 574 | 616.4 | 682.5 | 730.3 | 998.51 |
| 3 | 17 | 38.49 | 82.8 | 98.3 | 360.04 | 475 | 574.01 | 634 | 682.6 | 730.31 | 998.59 |
| 3.2 | 17.1 | 39 | 82.9 | 98.4 | 372.01 | 481 | 574.1 | 646.5 | 682.7 | 730.37 | 999.3 |
| 3.8 | 17.2 | 39.1 | 83 | 98.5 | 372.03 | 482 | 574.11 | 646.52 | 682.8 | 730.96 | 999.31 |
| 3.9 | 17.3 | 39.2 | 83.8 | 98.6 | 372.1 | 482.39 | 574.3 | 646.53 | 682.9 | 730.97 | V01.1 |
| 4 | 17.4 | 39.8 | 87 | 98.7 | 372.2 | 482.8 | 574.4 | 646.6 | 684 | 762.7 | V02.52 |
| 4.1 | 17.5 | 39.9 | 87.1 | 99 | 373 | 482.89 | 574.6 | 646.63 | 685 | 771 | V02.53 |
| 4.9 | 17.6 | 40 | 88.81 | 99.1 | 373.1 | 482.9 | 574.61 | 646.64 | 686.1 | 771.4 | V02.59 |
| 5 | 17.7 | 40.3 | 90 | 99.2 | 373.11 | 483 | 574.8 | 647 | 686.8 | 771.5 |  |
| 5.2 | 18 | 40.8 | 90.2 | 99.4 | 373.12 | 483.1 | 575 | 647.01 | 686.9 | 771.6 |  |
| 5.3 | 18.02 | 40.89 | 90.9 | 99.41 | 373.13 | 510 | 575.1 | 647.03 | 711 | 771.82 |  |
| 5.8 | 20.2 | 41 | 91 | 99.49 | 375 | 510.9 | 575.11 | 647.1 | 711.01 | 790.7 |  |
| 8 | 20.3 | 41.01 | 91.1 | 99.5 | 375.3 | 511.8 | 575.12 | 647.2 | 711.02 | 910.5 |  |
| 8.03 | 20.5 | 41.02 | 91.2 | 99.53 | 375.31 | 513 | 576.1 | 647.9 | 711.03 | 910.7 |  |
| 8.4 | 21 | 41.09 | 91.3 | 99.54 | 375.32 | 513.1 | 590.1 | 675 | 711.05 | 910.9 |  |
| 8.43 | 23 | 41.1 | 91.4 | 99.55 | 376.01 | 522.4 | 590.2 | 675.04 | 711.06 | 911.5 |  |
| 8.45 | 26 | 41.11 | 91.5 | 99.59 | 380 | 522.5 | 590.8 | 675.1 | 711.07 | 911.7 |  |
| 8.5 | 27 | 41.12 | 91.62 | 99.8 | 380.1 | 522.6 | 590.81 | 675.11 | 711.08 | 912.5 |  |
| 10 | 27.1 | 41.19 | 91.89 | 99.9 | 380.12 | 522.7 | 590.9 | 675.12 | 711.1 | 912.7 |  |
| 11 | 27.2 | 41.2 | 91.9 | 100 | 380.14 | 527.2 | 595 | 675.14 | 711.47 | 913.5 |  |
| 11.01 | 27.8 | 41.4 | 92 | 101 | 380.2 | 527.3 | 595.9 | 675.8 | 711.9 | 913.9 |  |
| 11.1 | 27.9 | 41.7 | 92.9 | 102 | 380.23 | 528.1 | 597 | 675.9 | 711.92 | 914.5 |  |
| 11.22 | 30 | 41.8 | 93 | 102.1 | 382 | 528.3 | 597.8 | 680 | 711.96 | 914.7 |  |
| 11.23 | 30.3 | 41.82 | 93.22 | 102.2 | 382.01 | 536.41 | 597.89 | 680.1 | 711.99 | 915.5 |  |
| 11.5 | 31 | 41.83 | 93.8 | 102.3 | 382.02 | 540 | 599 | 680.2 | 728 | 915.7 |  |
| 11.6 | 31.2 | 41.86 | 93.9 | 102.4 | 382.2 | 540.1 | 601 | 680.3 | 728.86 | 915.9 |  |
| 11.8 | 31.9 | 41.89 | 94 | 102.6 | 382.3 | 540.9 | 601.3 | 680.4 | 730 | 916.5 |  |
| 11.9 | 32 | 41.9 | 94.3 | 102.7 | 382.4 | 541 | 601.4 | 680.5 | 730.03 | 916.7 |  |
| 12 | 33 | 66.1 | 94.9 | 103 | 382.9 | 566 | 601.9 | 680.6 | 730.04 | 916.9 |  |
| 12.8 | 34 | 73 | 95.8 | 103.2 | 383 | 567 | 603.1 | 680.7 | 730.05 | 917.5 |  |
| 15 | 34.1 | 76 | 95.9 | 103.3 | 383.1 | 567.2 | 604 | 680.8 | 730.06 | 917.7 |  |
| 15.1 | 35 | 77 | 96 | 103.9 | 383.9 | 567.22 | 604.9 | 680.9 | 730.07 | 917.9 |  |
| 15.2 | 36 | 78.3 | 97 | 104.9 | 421 | 567.23 | 604.91 | 681 | 730.08 | 919.5 |  |
| 15.22 | 37 | 79.88 | 97.1 | 136.9 | 421.1 | 567.38 | 604.99 | 681.1 | 730.09 | 919.7 |  |
| 15.7 | 38 | 79.98 | 97.9 | 137 | 421.9 | 567.39 | 614.2 | 681.9 | 730.2 | 919.9 |  |
| 15.8 | 38.1 | 81.9 | 98 | 320 | 424.9 | 567.9 | 614.3 | 682 | 730.21 | 996.6 |  |

Covariate definitions

Pregnancy Diagnostic Codes (all subcodes included): V22, V23, V24, V28

HIV Diagnostic Codes (all subcodes included): 042

Cancer Diagnostic Codes (all subcodes included): any code between 140 to 239 (inclusive)
